# 657 A Novel Collaboration with Community Paramedics Impacts Length of Stay in a Burn Center

**DOI:** 10.1093/jbcr/iraf019.286

**Published:** 2025-04-01

**Authors:** Cassandra O’Rourke, Nicholas Asselin, Shea Amoriggi, Nelson Pedro, Kyle Jackson, Andrea Hernandez, Alicia Corey

**Affiliations:** Rhode Island Burn Center; Rhode Island Hospital and Brown University Healthcare; Lifespan; Rhode Island Hospital; Rhode Island Hospital; Lifespan; Lifespan

## Abstract

**Introduction:**

Burns require multifaceted management to facilitate hospital discharge. These complexities with limited home care resources contribute to discharge delays and potentially increase Hospital Length of Stay (HLOS) in this population. We describe the impact of a Mobile Integrated Healthcare/Community Paramedicine (MIH/CP) partnership on HLOS in a Burn Center.

**Methods:**

This study included patients admitted to an adult/pediatric burn center with a diverse catchment area, over a 54-month period. A retrospective study of burn registry data comparing a cohort of admitted patients with burn injuries from January 2020-March 2022 (pre-implementation, n= 392 with a similar 27-month cohort from April 2022-July 2024 with and without MIH/CP services (MIH/CP, n= 70, no MIH/CP n= 383. Exclusions included patients who were deceased, transferred to other facilities, or discharged to skilled nursing/rehab. the final cohorts were pre-implementation (n= 329), and post-implementation MIH/CP (n= 69) and no MIH/CP (n= 303). Data included TBSA, hospital LOS (HLOS) and planned and unplanned readmission rates from the burn registry. These were matched with operational data from the MIH/CP program including: Time on service and number of visits. The primary outcome was the HLOS/TBSA rate among the groups with secondary outcomes of planned and unplanned readmissions. Data were analyzed using StatPlus/Microsoft Excel and reported as median [IQR], p values are for Mann-Whitney U tests and Chi2 tests.

**Results:**

For the primary outcome of HLOS/TBSA rate, there was no difference between the pre & post-implementation cohorts (1 vs 1.2, p=0.37). The groups were similar in terms of age, gender, TBSA. For the secondary outcome of planned and unplanned readmissions, the rates were similar between groups (planned 7.3% vs. 8.9% p= 0.45 and unplanned 4.3% vs 4.3% p=0.98). In the post implementation period separating those with and without MIH/CP there was a non-significant (p=0.25) trend towards a lower HLOS/TBSA rate in the MIH/CP group (1.12 vs. 1.33). Cohorts also differed in age (MIH/CP 45 vs no MIH/CP 35, p=0.05), TBSA (5 vs. 2.8, p< 0.001) and LOS (5 vs 3, p< 0.001). Unplanned (8.7% vs. 3.3% p=0.05) and planned (22% vs. 6% p=< 0.001) readmission rates were also higher in the MIH/CP cohort when compared with the no MIH/CP group.

**Conclusions:**

A novel collaboration between a burn center and MIH/CP was feasible and resulted in more complex (older, higher TBSA) patients being cared for by MIH/CP providers. Although not significant, despite the increased complexity of cases, the MIH/CP cohort had lower HLOS/TBSA rates when compared with patients not referred to MIH/CP. These data suggest that MIH/CP is an effective means to care for complex burn patients in the community.

**Applicability of Research to Practice:**

Future data can be utilized to determine financial implications of decreased HLOS, increased referrals to PT/OT and impact on outpatient clinic volume and revenue.

**Funding for the Study:**

N/A

